# A Novel EEG Based Spectral Analysis of Persistent Brain Function Alteration in Athletes with Concussion History

**DOI:** 10.1038/s41598-017-17414-x

**Published:** 2017-12-08

**Authors:** Tamanna T. K. Munia, Ali Haider, Charles Schneider, Mark Romanick, Reza Fazel-Rezai

**Affiliations:** 1 Department of Electrical Engineering, University of North Dakota, Grand Forks, 58202 USA; 2 Department of Physical Therapy, University of North Dakota, Grand Forks, 58202 USA

## Abstract

The neurocognitive sequelae of a sport-related concussion and its management are poorly defined. Detecting deficits are vital in making a decision about the treatment plan as it can persist one year or more following a brain injury. The reliability of traditional cognitive assessment tools is debatable, and thus attention has turned to assessments based on electroencephalogram (EEG) to evaluate subtle post-concussive alterations. In this study, we calculated neurocognitive deficits combining EEG analysis with three standard post-concussive assessment tools. Data were collected for all testing modalities from 21 adolescent athletes (seven concussive and fourteen healthy) in three different trials. For EEG assessment, along with linear frequency-based features, we introduced a set of time-frequency (Hjorth Parameters) and nonlinear features (approximate entropy and Hurst exponent) for the first time to explore post-concussive deficits. Besides traditional frequency-band analysis, we also presented a new individual frequency-based approach for EEG assessment. While EEG analysis exhibited significant discrepancies between the groups, none of the cognitive assessment resulted in significant deficits. Therefore, the evidence from the study highlights that our proposed EEG analysis and markers are more efficient at deciphering post-concussion residual neurocognitive deficits and thus has a potential clinical utility of proper concussion assessment and management.

## Introduction

A concussion is a complex pathophysiological procedure which is induced by sudden impulsive biomechanical forces affecting the brain^1^. In the US alone, sport and physical activity cause nearly 4 million concussions each year^2,3^. It is critical to assess concussion and mild traumatic brain injury (mTBI) with high accuracy to avoid anxiety, sensitivity and cognitive biases which appear as post-concussion syndrome. Moreover, insufficient follow-up and treatment can put the post-concussive person at the risk of neurobiological depression with anxiety resulting in a longer concussion recovery time. Therefore, proper understanding and measuring of concussions are essential to treat the psychological factors as a means of effective prevention which, in turn, can lead to a rapid post-concussion recovery period. When examining performance metrics related to motor control, it is well established that individuals diagnosed with the post-concussion syndrome can show marked impairments in reaction times^4^, visual motor processing^5^, gait stability^6^, postural balance^7^ and dynamic gait analysis^8,9^. More importantly, it is a primary concern for both amateur and professional athletes. Because the symptoms of concussions sometimes go unnoticed or are self-reported and tend to subside within 1–2 weeks^10^, many athletes fail to seek immediate and proper medical care. Furthermore, high school athletes tend to purposely avoid reporting their concussions in order to prevent being “benched” during subsequent games^11^. Though almost all recreational participants express their concern about post-concussion syndrome, most competitive athletes keep quiet about their minor physical discomforts or even deny considerable pain for the sake of pursuing their career goals. Although athletes’ willingness of accepting risks greatly varies with the competition stages, game completion levels and types of sports, it’s more likely that many individuals will choose to continue to play with a concussion rather than remove themselves from competition^12^. However, such a decision can pose a risk to their health with the potential for repeated head trauma^13^. Athletes have been shown to suffer from cognitive deficits up to three years after their brain injury incidents, exhibiting lower performance on select neuropsychological tasks when compared to an age-matched non-concussed group^14^.

Evidently, the challenges in concussion assessment have led to the studies exploiting the sensitivity of EEG spectral features to mild, moderate, and severe traumatic brain injury over the time span as short as 15 days to four years post-concussion.

Researchers have accomplished the quantitative analysis of the EEG signals collected from the concussed subject to evaluate the post-concussion physical and clinical recovery. Additional studies suggest that the EEG spectral profile varies with acute mTBI due to the change in the cognitive state during the resting stage^15,16^. In essence, the spectral profile of EEG is also altered in acute mTBI and during any anomaly of consciousness. However, researchers argue whether mTBI can evoke long-term variations in spectral information. Also, identification of any long-term change is sometimes controversially attributed to psychiatric comorbidity such as posttraumatic stress disorder (PTSD). So far, long-term neurological changes have remained indistinct. Nevertheless, many findings support that brain volume, and white matter can be affected by mTBI^17^. Likewise, the resting state activation stage can be sensitive to mTBI. Another study found that EEG measurement was able to predict the return to play better than other measurement types^18^. Notably, one study examined EEG and showed that frequency information changes for as long as six months after the mTBI occurrence^19^. All these findings underscore the fact that the power of each frequency component of EEG can reveal significant physiological and clinical findings. Though there is a necessity to examine the details of spectral patterns after a mTBI incident, only a relatively small number of studies compared the spectral profiles just with a group of frequencies bounded by specific bands.

The goal of the current research is to look into the spectral profiles as a potential measurement tool which can expose the long-term cognitive impairment after an analytical study of EEG signals. To test our hypothesis, we utilized visual (King-Devick (K-D) Test), postural (Balance Error Scoring System (BESS)) and neurological (Immediate Post-Concussion Assessment and Cognitive Testing battery (ImPACT)) tests, along with a novel EEG spectral analysis that computes the distinguishing features from each individual component of EEG, as well as from the set of conventional frequency bands. We also utilized novel time and nonlinear feature-based analysis to evaluate the EEG of injured and healthy athletes that provide unique and complementary measures of post-concussion deficiencies. Herein, we report that though postural, visual and neurological tests were unable to detect the deficits associated with a long-term concussion history, the EEG linear and nonlinear feature based spectral analysis, both in terms of frequency bands and individual frequencies, were sensitive to highlight post-concussion sequelae.

## Methods

### Participants

The inclusion criteria for the participants were adolescents high school athletes aged 14–18 years who were actively participating in football games. Adolescent athletes were emphasized in this study since according to CDC report, youths are at increased risk of concussion, and 65% of these concussions occur in children between 5 to 18 years of age^20^. These persons are at a larger risk for traumatic brain injury as their brains are still young and developing, and the brain tissues are not as able to recover as rapidly as an adult brain^21^. The data collection was limited to football to align with the highly broadcasted wave of concerns about the sport-related brain trauma in National Football League (NFL) stars. Exclusion criteria for the participants included any history of intellectual or learning disabilities, neurological or psychotic disorders, or alcohol/substance abuse.

Following the inclusion and exclusion criteria, we were able to collect data from a total of 21 male participants who are football athletes from two high schools available in Grand Forks area. The study was performed following the experimental protocol approved by the Institutional Review Board (IRB) of the University of North Dakota. The data were collected in accordance with the guidelines and regulation established by the protocol. The participation was voluntary, and the participants had the right to withdraw any time from the study. Informed and written consent for participation was collected from the athletes and also from their parents or legal guardians. Each participant had to complete a demographic information form with previous concussion history before data collection.

Individuals recruited for this concussion analysis study were assigned to a particular group based on the history of concussion. The healthy group consists of 14 subjects (Age 15.86 ± 0.67 years, Height: 1.75 ± 0.09 m, Weight: 72.82 ± 10.03 Kg) with no history of concussion while the concussed group has 7 subjects (Age 15.97 ± 0.74 years, Height: 1.77 ± 0.09 m, Weight: 73.20 ± 12.56 Kg) who suffered from one or multiple previous concussions. The concussion was detected by the concussion management team (including athletic trainer and team physicians) assigned by the respective schools who was present on the sideline during the athletic contest. The concussion management team detected the concussion by following the established criteria suggested by American Academy of Neurology Guideline for Management of Sports Concussion^22^ and state law of North Dakota^23^.

All participants were actively participating in sport and athletes with concussion history made a complete return to play within four weeks of injury. All athletes with a history of concussion (12 days to 15 months from injury) reported being symptom-free at the time of testing. Control participants were teammates who had never suffered a sport or non-sport related brain injury. Concussed participants’ post-concussion status is shown in Table 1.

**Table 1:** Concussed participants demographic information.

Concussed Participants	Number of concussion	Loss of consciousness	Confusion	Amnesia	Post-concussion RTP days	Days from concussion incident to data collection		
						From incident 1	From incident 2	From incident 3
1	2	No	Yes	Yes	14	263	216	—
2	1	No	Yes	Yes	21	118	—	—
3	1	No	No	No	7	267	—	—
4	3	No	Yes	Yes	10	462	297	162
5	2	No	Yes	Yes	25	92	65	—
6	1	No	No	No	10	127	—	—
7	1	No	No	Yes	15	12	—	—

From each subject, the traditional assessment data and EEG signals were collected in three different trials with 30-days’ time difference between the trials. The total number of data collection trials for the healthy group was multiplied by fourteen (total 42 trials) and for the concussed subject was three multiplied by seven (total 21 trials).

### Postural Data Collection Protocol

#### Balance Error Scoring System (BESS)

The BESS is one of the most popular tests used to find balance deficit in concussed and fatigued athletes^24^. Postural stability is measured using three stances, named double leg stance, single leg stance, and tandem stance. Each test is done on two different surfaces, first on a firm surface and then on a foam surface. During these stances, athletes’ eyes are closed, and their hands are placed on the iliac crests and feet positions are different based on three distinct stances. Each of these six subtests is performed for 20 seconds. Deviation from proper stance is referred to an error, and the total number of errors during the subtests are counted.

### Visual Data Collection Protocol

#### King-Devick (K-D) Test

The K-D test is a test of the visual system and is based on measurement of the speed of rapid number naming^25^. The K-D test is faster than other standardized tests like ImPACT, Military Acute Concussion Evaluation (MACE) and the sports concussion assessment tool (SCAT 3) as it takes just two minutes to complete the testing and thus is more practical in case of sideline application^25^. The K-D test consists of three test cards and the athlete’s need to name the numbers from the cards rapidly without any error. The score for the test is calculated by combining the amount of the three times, in seconds, required to read the three cards. The test involves attention, rapid eye movements as well as language operation. These three functions may be adversely affected, resulting in a poor K-D test performance. The test purports to measure any suboptimal brain functional deficits after a concussion incident, as well as sometimes reflects deficits due to sleep deprivation, Parkinson’s disease, hypoxia and multiple sclerosis. In this experiment, we performed the K-D test to find out the efficacy of this test to assess suboptimal brain functional deficit due to a concussion after a time gap between concussion incident and data collection.

### Neuropsychological Data Collection Protocol

#### Immediate Post-Concussion Assessment and Cognitive Testing (ImPACT)

The ImPACT battery is the most common computerized test that can be used in cognitive concussion assessment^26^. The test battery consists of three different measures: Demographic data, neuropsychological tests, and the Post-Concussion Symptom Scale (PCSS). The assessment results from these three sections are combined to assist in accurate evaluation and management of concussion^27^. The demographic data section mainly consists of all the important sport, medical, and concussion history related information. For the neuropsychological test sections, ImPACT (version 3.0) contains six different neuropsychological tests, and each of these tests is intended to target different parts of cognitive functioning comprising attention, verbal and visual memory, control, reaction time and processing speed. Combining the results from these six different tests, a set of composite scores are produced containing separate measures named verbal memory, visual memory, motor speed, reaction time and impulse control. The detailed description of these tests can be found at^26–28^. The last section named PCSS is also utilized in the ImPACT battery study^28^. The scale is reported by various sports organizations to manage and track post-concussion symptoms^26–29^. This section has a 21-symptom checklist which mainly asks the athlete to specify a rate for each symptom on a scale of one to seven, with zero representing no presence of a symptom and six representing a severe symptom. An ImPACT test was performed by all participants during all three trials.

### EEG Data Collection Protocol

**Figure 1:**
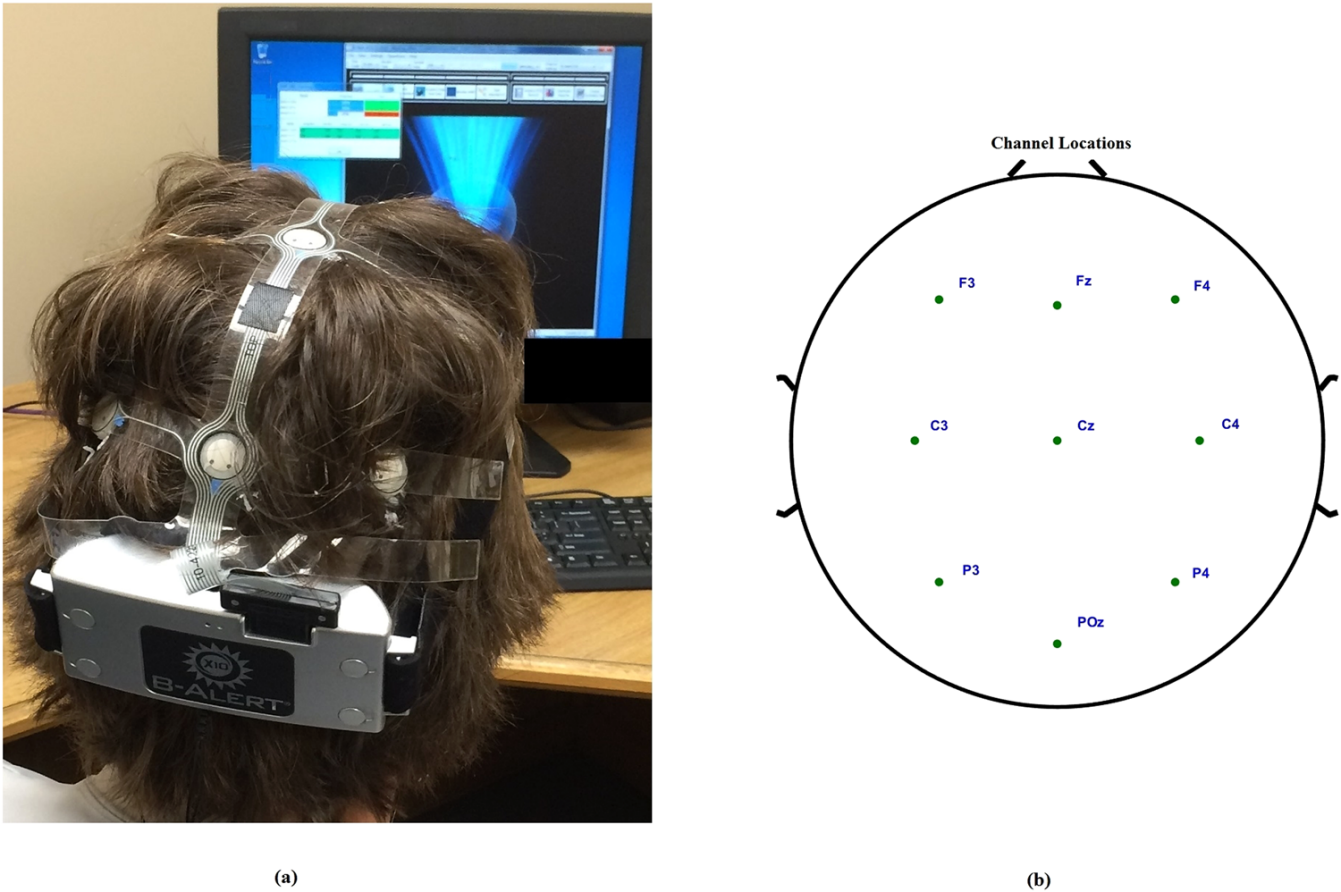
Experimental setup for EEG data collection. (**a**) Data collection set up for a participant, (**b**) Map of 9 Electrodes locations. The locations were plotted using EEGLAB^31^.

EEG activities were measured using a 9-lead wireless B-Alert headset^30^. Electrode impedance was kept below 50 kΩ. During data collection, the left mastoid was used as a reference, and the right mastoid was used as a ground. The sampling rate for data collection was 256 Hz, and data were acquired by placing nine electrodes at F3, F4, Fz, C3, C4, Cz, P3, P4 and POZ locations as shown in Fig. 1.

The data were collected for 5 minutes from all 21 subjects during different trial sessions each under three conditions: vigilant task (VT), eyes open (EO), and eyes closed (EC). During VT condition, the subject was highly engaged by choosing between a primary vs. secondary or tertiary task every 1.5 to 3 seconds. During EO condition, the subject goes through a low engagement state by responding to an optical probe every 2 seconds. The EC state creates a distraction status, and the subject has to respond to an audio tone every 2 seconds. The same procedure was followed at all different trials for all subjects.

### **EEG Data Analysis**

The EEG data were first high pass filtered above 1 Hz and then low-pass filtered below 40 Hz, and thus a 1–40 Hz (24 dB/octave) band-pass filter was formed. The first and last 10 s of each 5-min recording during EO, EC, and VT conditions were rejected to eliminate state transitions. The EEG data were then visually inspected to determine clean EEG data and randomly occurring large amplitude with power ≥ 3 standard deviations with respect to the mean value of the clean EEG was removed. Afterward, the stereotypical noise like eye movements, eye blinks, muscular activity, line noise, motion related signal, and heart signals was cleaned by using well-established Independent Component Analysis procedure of EEGLAB detailed previously^31,32^. Any other nonstereotyped or residual artifact was removed through visual inspection of the raw data.

The clean EEG data was then segmented into 1-second epochs containing 256 data points. Power spectral density (PSD) was determined by computing Fast Fourier Transformations (FFT) with a 10% Hanning window on each segment to determine spectral power (μV^2^) for 1 to 40 Hz frequency bins of each EEG channels. The PSD of the individual bins were then averaged and logged to calculate PSD of conventional EEG frequency bands named delta (1–4 Hz), theta (4–8 Hz), alpha (8–12 Hz), beta (12–30 Hz) and gamma (30–40 Hz). Although gamma band is considered as a pattern of neural oscillation with a frequency between 30 Hz to 100 Hz nowadays, we have used 30–40 Hz as gamma band in this study since the gamma band was previously defined up to 40 Hz^33^. Numerous studies reported that the gamma band is most apparent at the frequency of 40 Hz^34–38^. While most gamma oscillation study emphasized frequencies around 40 Hz, electrocorticographic recordings (ECoG) in patients enduring epilepsy have suggested that the functional activation may be more consistently connected with the higher frequencies, typically greater than 60 Hz and may extend up to 200 Hz and beyond^39^. In our study, the 30 Hz to 40 Hz was chosen as gamma band since all the analysis of this study is based on EEG and also the interpretation of the earlier EEG based concussion studies suggested that the EEG spectrum contains some characteristic waveforms associated with concussion which primarily fall within the frequency band of 1 to 40 Hz^15,40–47^. Moreover, the use of high-frequency gamma band is still controversial as studies showed that the change in the higher frequency gamma-component might be a result of the higher amount of artifact from the electromyographic activity^48–50^.

After calculating the PSD for each channel and bands, overall PSD was calculated by calculating the mean PSD across all nine referential channels for both individual frequency bins and five frequency bands. Linear and nonlinear features were then extracted from the five frequency bands and also from each of 1 to 40 Hz EEG frequency bins.

This innovative analysis achieved a new range of frequencies with significant differences between healthy and concussed groups even when the band base analysis was not adequate to reveal the deficits. Moreover, in this paper, we present an exploration of the usefulness of several features for use in concussion detection, which aims at providing accurate feedback as early as possible. Along with the traditionally used band power estimates, we computed some time domain as well as nonlinear features from each EEG frequency band and then again computed all the features from each individual frequency bins. The parameters extracted from EEG signal are explained as follows.

#### Linear Features

Power spectral density analysis was performed to extract the linear features from the signal. The extracted features were; (i) average spectral power for five frequency bands and (ii) the spectral power for each of the individual frequency from 1 Hz to 40 Hz.

#### Time domain Feature

Most popular features used for concussion analysis are EEG band based power spectral density. In this paper, we introduce new features for concussion assessment called Time Domain Parameters that are also known as Hjorth parameters. The features are inspired by the fact that they have been previously used in EEG based experiments like Vidaurre *et al*. used Hjorth parameters, in their brain-computer interface (BCI) study^51^ whereas Cecchin *et al*. used Hjorth parameters for seizure assessment from raw scalp EEG signals^52^. The parameters introduced by Hjorth^53^ are three features defined as follows by equation 1 to 3:

1 $$Activity\,(x(t))=var(x(t));$$

2 $$Mobility\,(x(t))=\sqrt{\frac{var\,(\frac{dx(t)}{dt})}{var\,(x(t))}}\,;$$

3 $$Complexity\,(x(t))=\frac{Mobility\,(\frac{dx(t)}{dt})}{Mobility\,(x(t))}$$

The first parameter, Activity, calculates the alteration of time signal and characterizes the signal power. Mobility is computed by calculating the square root of the variance of the first derivative of the signal divided by the activity and thus specifies the average frequency or proportion of standard deviation of the spectral power. Complexity describes the change in frequency by comparing the Mobility of the first derivative of the signal with the signal’s mobility, and for more resemblance between the signals, the value converges to one. These three parameters consider the frequency component of the signal itself and thus remain more robust against the errors due to overfitting or non-stationarities of the signal^52^. To reduce the complexity of calculation, these three parameters were calculated in a stationary mode of signal separately for each EEG channel of the entire signal. Thus, the extracted parameters were three features per channel and, as a whole, a feature vector for each parameter. A total of 27 features (3 features for nine channels) were extracted and then averaged for all channels.

#### Nonlinear Features

Different nonlinear parameters have been shown significantly useful in the diagnosis of neurological disorders. Nonlinear parameters like approximate entropy (ApEn), Hurst exponent, and Correlation dimension have been used for automatic diagnosis of seizure onset and reported as a promising approach in differentiating normal, pre-ictal and epileptic seizure from EEG signals^54^.

In the field of cortical neuronal dynamic study, the existence of long-range temporal correlation (LRTC) is considered a potential observed phenomenon as it is proven to be gradually reduced with the power-spectrum^55^. The LRTC property of an amplitude-time signal has vital importance as it is found to have a relationship with the distributed neural network^33^. Poil *et al*. reported the coexistence of LRTC property of amplitude time series with neuronal avalanche activity^56^, and thus recommended a relationship between oscillatory activity detected in the EEG and the criticality hypothesis^56,57^. Using these hypotheses, Shew *et al*. suggested a possible connection between optimal functioning and LRTC in the amplitude of oscillations^35^. Moreover, the significance of the LRTC property has also been proven in numerous clinical studies linking a number of neuronal diseases (including schizophrenia^58^, Alzheimer’s disease^59^, major depressive disorder^60^, and epilepsy^61^) with altered LRTC properties. To quantify the degree of change in LRTC property in a signal, the Hurst exponent (H), (explained in a later paragraph) is measured^55^. Hurst exponent was used by Holler *et al*. for the disorder of consciousness studies^62^ whereas Culic *et al*. reported this property to be important to differentiate epileptic patients^63^.

Another nonlinear parameter that was calculated was ApEn. ApEn is a widely known mathematical algorithm which computes the predictability of time series data by quantifying the regularity and complexity of the signal. ApEn quantifies the logarithmic likelihood of the patterns in the signal that remain close on next incremental comparisons^64^.

Values of the ApEn parameter have been reported significantly different between EEGs collected from epileptic seizure patients and normal EEG signals^65^. Guo *et al*. present a method based on approximate entropy for classifying the EEG regarding the existence and absence of seizures using the neural network with 99.85% accuracy^66^.

Inspired by these publications, we tested the efficacy of these features to distinguish healthy and concussed athletes in this study. Approximate entropy (ApEn) and Hurst exponent were extracted as the nonlinear features to measure synchrony and complexity of the EEG signal as explained in the following sections.

#### Approximate Entropy

ApEn was calculated for each frequency (1 to 40 Hz) and for each of five frequency bands of EEG data for all three different conditions in order to find out if there was any relationship between the randomness of EEG data along with a concussion. A lower value of approximate entropy specifies that the EEG data is more deterministic whereas a higher value of ApEN determines the data is more random. This feature was calculated using the ApEn function provided by Kijoon Lee in the MATLAB central file exchange^67^. The tolerance chosen for ApEn calculation was two standard deviations.

#### Hurst Exponent

The Hurst exponent (H) calculates the extent information presented by a signal is related to the history of the signal. The value of H varies from 0 to 1; 0 < H < 0.5 indicates the samples in the signals are far apart and independent and thus the signal is short-range dependent. However, if 0.5 < H < 1, then the value is said to contain LRTC, with higher values of H representing a stronger LRTC property^55^. The Hurst exponent is thus known as the index of long-range dependence^68^. The value of H was calculated for each channel over the entire EEG signal. A total of 9 components for 9 EEG channels were extracted for each signal.

### Statistical Analysis

The deficits between healthy and concussed groups were verified using statistical analysis, and the measurements were performed without knowledge of groups. The Shapiro-Wilk test was applied to ascertain the normality of the data. For normally distributed data, a two-tailed Student t-test, followed by Bonferroni’s post hoc test when applicable was implemented; otherwise, Wilcoxon rank sum test was considered. The values in the manuscript are presented as mean ± standard deviation format with statistical significance level set at (p = 0.05). The test of significance was performed using the MATLAB Statistical Toolbox^69^.

### Data Availability

The database generated and/or analyzed during the current study will be available from the corresponding author upon request.

## Results

### BESS Test

Postural deficits in terms of the BESS associated with concussion showed no significant difference between healthy and concussed group. Average sway per second was calculated using a modified Wii balance board during the BESS assessment for healthy group (group average sway = 3.28 ± 0.69 cm) and concussed group (group average sway = 3.00 ± 0.72 cm). The number of average BESS errors reported by the healthy group were thirty compared to thirty-four reported by the concussed group. Though the average sway scores exhibited by both groups were quite similar, the concussed group reported more errors than their healthy matched controls. The t-test resulted in no significant differences (Average sway: *p-*value* = *0.33, Number of errors: *p-*value* = *0.39) between the groups regarding average sway and number of errors.

### K-D Test

K-D test measures the deficiencies of attention and eye movements by capturing the speed of rapid number naming. The athletes who sustained concussions required slightly more time to complete the task than their peers in the healthy group (by approximately 0.1%), but the deficits did not reach a level of significance (Healthy group: 53.20 ± 10.33, Concussed group: 53.74 ± 10.29; *p-*value = 0.966).

### ImPACT Test

The healthy and concussed groups were not significantly different with regard to age but were significantly different based on the number of prior concussions. A two-tailed t-test was performed to evaluate the differences in neuropsychological test performance regarding ImPACT battery between the concussed and control groups. Table 2 presents the detailed descriptive statistics for verbal and visual memory, processing speed, and reaction time composite scores.

**Table 2:** Group means and standard deviations for ImPACT composite scores of healthy and concussed groups. The test of significance was performed with statistical significance level of 0.05.

Composite Scores	Healthy Group	Concussed Group	F value	*p*-Value
	Mean ± SD	Mean ± SD		
Verbal Memory Index	89.93 ± 7.87	87.57 ± 9.25	0.58	0.58
Visual Memory Index	86.43 ± 5.37	82.57 ± 7.79	0.25	0.27
Motor Speed Index	40.36 ± 5.81	37.28 ± 5.37	0.75	0.20
Reaction Time Index	0.62 ± 0.09	0.65 ± 0.12	0.38	0.59
Impulse Control Index	5.86 ± 3.21	6.14 ± 2.9	0.86	0.84
Total Symptom Score Index	2.93 ± 2.13	3.14 ± 3.53	0.12	0.88

Though a number of studies reported the ability of the ImPACT to differentiate healthy and concussed groups, our analysis revealed no significant difference in any composite scores between the groups.

### Neuronal Deficits in Terms of EEG Band-Power following Concussion

The EEG analysis was conducted to extract the neuronal deficits following a concussion. Athletes in the concussed group exhibited an increase in delta and theta bands, and a decrease in alpha, beta and gamma frequencies compared to their uninjured peers during all three testing conditions (Table 3). As indicated in Table 3, the difference reached the significance level for the increase in delta band and decreased in alpha, beta and gamma frequency bands for all three conditions.

**Table 3:** EEG band power deficits between healthy and concussed group for eyes open (EO); eyes closed (EC) and vigilant task (VT) conditions. (*Denotes significant differences between healthy and concussed group at statistical significance level of 0.05).

Condition	Participants	Delta (μV^2^)	Theta (μV^2^)	Alpha (μV^2^)	Beta (μV^2^)	Gamma (μV^2^)
		Mean ± SD	Mean ± SD	Mean ± SD	Mean ± SD	Mean ± SD
EO Condition	Healthy	4.33 ± 0.25	3.47 ± 0.33	3.14 ± 0.36	2.48 ± 0.30	1.95 ± 0.15
	Concussed	4.81 ± 0.34*	3.67 ± 0.38	2.69 ± 0.25*	2.14 ± 0.18*	1.58 ± 0.21*
EC Condition	Healthy	4.21 ± 0.33	3.43 ± 0.28	3.22 ± 0.23	2.46 ± 0.26	1.92 ± 0.13
	Concussed	4.66 ± 0.30*	3.59 ± 0.33	2.85 ± 0.34*	2.13 ± 0.33*	1.50 ± 0.34*
VT Condition	Healthy	4.22 ± 0.24	3.38 ± 0.49	3.09 ± 0.40	2.47 ± 0.27	1.97 ± 0.17
	Concussed	4.68 ± 0.45*	3.58 ± 0.34	2.65 ± 0.28*	2.08 ± 0.29*	1.52 ± 0.21*

### Neuronal Deficits in Terms of EEG Individual Frequency Power following Concussion

**Figure 2:**
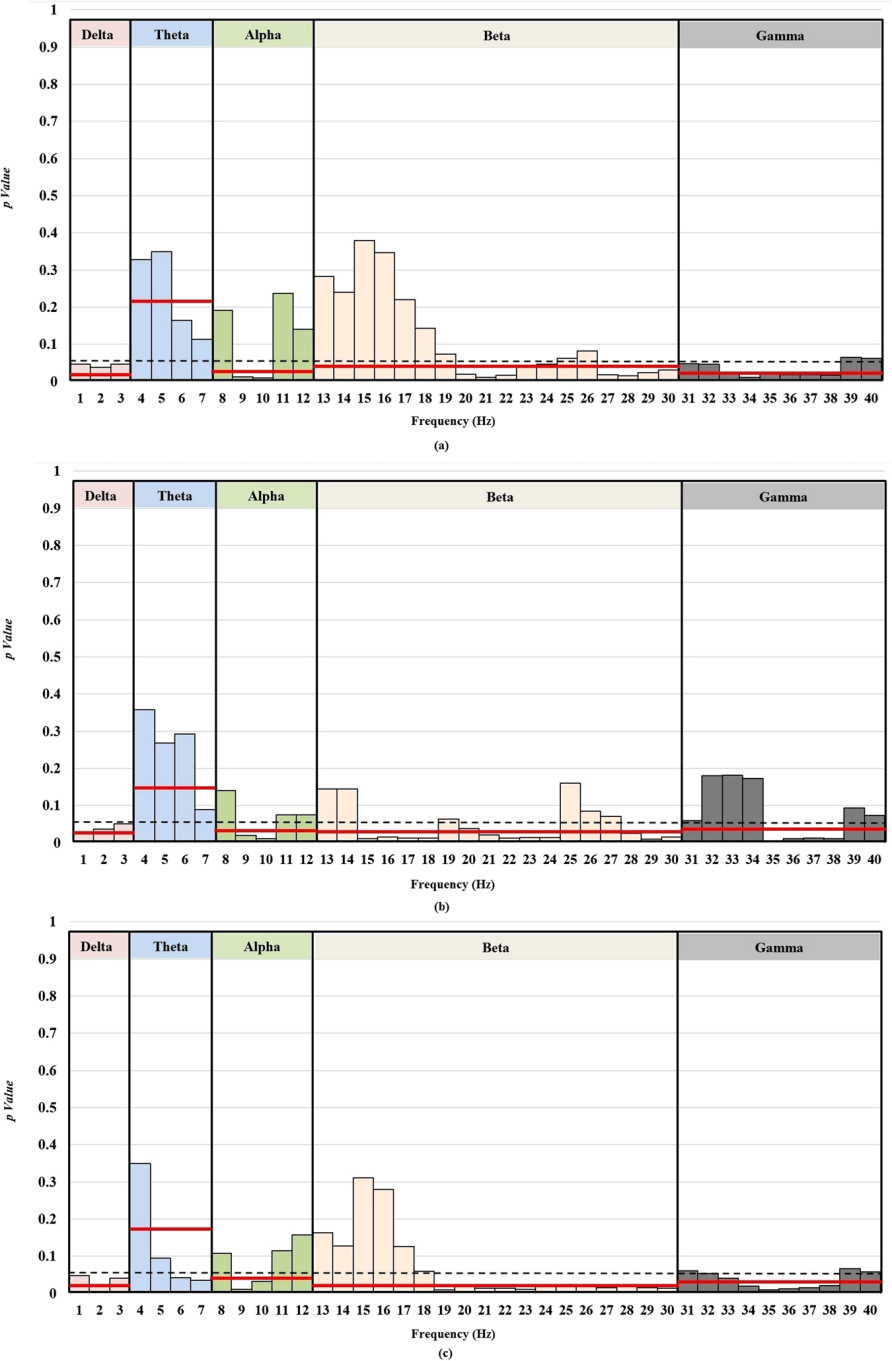
*P*-value vs. frequency plot. A set of individual frequencies from EEG data exhibits power spectral density deficits between healthy and concussed athletes. The X-axis in the figure shows the individual frequencies and Y-axis shows the level of significance. The color of bars is different based on each frequency band, and the level of significance for each EEG frequency band is shown by red lines. The *p*-value vs. frequency is shown during three conditions (**a**) eyes open (EO) (**b**) eyes closed (EC), and (**c**) vigilant task (VT). All the test of significance was performed with statistical significance level of 0.05.

This analysis considered individual EEG frequencies to find gaps between healthy and concussed groups. Figure 2 shows the results of both frequency band and individual frequency analysis for three experimental conditions (EO, EC, and VT). The dashed black line shows the confidence level of *p* = *0.05*. The solid red lines show the *p*-value for each frequency band (delta, theta, alpha, beta, and gamma bands). The bars in each frequency band show the *p*-value for individual frequencies. To highlight the subject to subject variance for each group, a supplementary table with the mean and standard deviation of power of each significant frequency bins for each group is added to the manuscript.

The athletes who sustained a concussion had a range of frequencies with a significant difference from the healthy group during EO condition (1–3 Hz, 9–10 Hz, 20–24 Hz, 27–30 Hz, and 33–38 Hz) as shown in Fig. 2(a). A very similar, but not all range of significance was exhibited during EC condition (1–3 Hz, 9–10 Hz, 15–18 Hz, 20–24 Hz, 28–30 Hz, and 35–38 Hz) as shown in Fig. 2(b). The significant individual frequencies exhibiting the deficits between healthy and concussed groups during VT condition were (1–3 Hz, 6–7 Hz, 9–10 Hz, 19–30 Hz, 34–38 Hz) and were much consistent with EO condition as shown in Fig. 2(c).

### Neuronal Deficits in terms of Nonlinear Features from EEG Individual Frequency following Concussion

**Figure 3:**
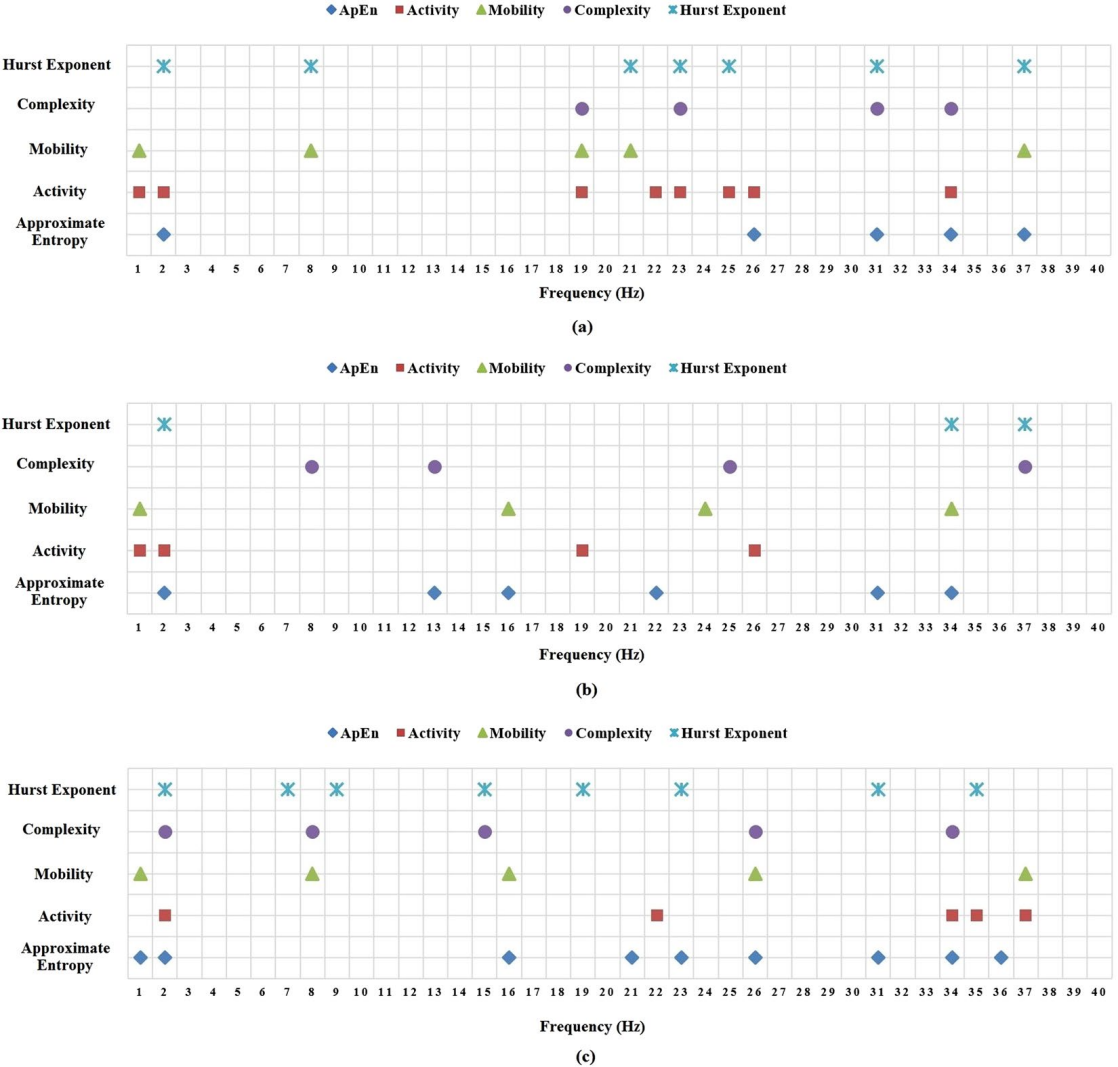
Frequencies with a significant difference in approximate entropy, activity, mobility, complexity and Hurst exponent between healthy and concussed athletes for three conditions: (**a**) eyes open (EO), (**b**) eyes closed (EC), and (**c**) vigilant task (VT). All the test of significance was performed with statistical significance level of 0.05.

In the final analysis, nonlinear features were calculated in order to find out if new features extracted from the EEG data can tabulate the deficiencies due to a concussion. The extracted features were approximate entropy, activity, mobility, complexity and Hurst exponent features. While calculating these features for EEG frequency bands (delta, theta, alpha, beta, and gamma), no significant deficits were found between healthy and concussed athletes. But when the analysis was done for individual frequencies instead of frequency bands, interesting outcomes were exhibited. A set of individual frequencies was found for each nonlinear feature which can reveal significant deficits between healthy and concussed athletes as reported in Fig. 3. As shown in Fig. 3(a) for EO condition, the frequencies indicating significant deficits between healthy and concussed groups in terms of 2 or more nonlinear features are 1–2 Hz, 8 Hz, 19 Hz, 21 Hz, 23 Hz, 25–26 Hz, 31 Hz, 34 Hz and 37 Hz. For EC condition, Fig. 3(b), the range of frequencies with deficits in two or more features was for 1–2 Hz, 13 Hz, 16 Hz, 34 Hz and 37 Hz, and for VT condition, from Fig. 3(c), the range was 1–2 Hz, 8 Hz, 15–16 Hz, 23 Hz, 26 Hz, 31 Hz, 34–35 Hz and 37 Hz. The most efficient nonlinear features to reveal deficiency following concussion were approximate entropy, activity and Hurst exponent feature.

## Discussion

Residual damage to the brain due to concussion can often evade clinical detection. Enhancing ways in which concussion is assessed is pivotal, specifically in susceptible individuals such as adolescent athletes where functional deficits can be elusive and seriously underreported. Better assessment is also essential since early identification of the signs of a concussion can progress positive outcomes and thus suggests that there is a clear need for an effective evaluation approach to efficiently assess and quantify high-risk individuals such as athletes who may have already sustained a concussion. The current study aims to test the hypothesis that the concussion disrupts the normal brain activities of a person. To detect these deficits, we combined the BESS, K-D test, ImPACT, and EEG analysis to capture the postural, suboptimal, neurophysiological and neuronal deficits following a concussion.

Evidence from the previous studies^29,40,70^ shows that the cognitive impairment regarding the BESS is most pronounced during the time of injury and 24 hours post injury but appears to resolve by day five after a concussion incident. The balance deficit through the BESS in our research resulted in no significant difference between the healthy and concussed group and thus strengthened the already established hypothesis^26,40,70^ that the postural deficits resolve within a brief period post-injury and therefore may suggest that the BESS is not sensitive enough to interpret any residual deficits associated with long-term concussion history.

As expected, the K-D test, which is mainly a rapid screen tool and typically used immediately after concussion^25^, was unable to detect any deficits in our study. This can be explained by the fact that the related visual deficits due to a concussion were resolved during the several months’ time gap between the concussion incident and data collection.

The ImPACT was reported by multiple sports-related concussion studies as a potential tool to detect the impaired neurocognitive functioning due to concussion^26–28^. Also, some studies showed neuropsychological baseline assessment models like ImPACT could assist the diagnosis of subtle neurocognitive deviations in athletes after a concussion incident^26,27^. Though several studies demonstrated that a history of concussion is associated with poorer performance in ImPACT^71^, the role of concussion history remains a controversial issue, with various studies yielding no relationship between concussion history and ImPACT performance^28^. The results of this manuscript suggest that there is no significant effect of a history of concussion associated with performance measured by ImPACT, which is understandable, as ImPACT is an immediate post-concussion paradigm, and due to the long time gap between concussion incident and data collection, the sensitivity of the test deteriorates with time.

To capture the signature neuronal deficits exhibited by concussed athletes that distinguish them from their healthy peers, we evaluated several approaches utilizing a set of linear, time-frequency based features along with nonlinear features extracted from EEG signals. In conjunction with band base analysis, this study undertook a systematic exploration to find out the deficits within specific frequency bins from 1 to 40 Hz. The system works by following four main steps: data acquisition, data preprocessing, feature extraction (power spectral, time domain and nonlinear) and statistical analysis (functional deficits detection).

For band base analysis, EEG was divided into traditional frequency bands (delta, theta, alpha, beta, and gamma). After normalization, power spectral density analysis revealed a significant difference between healthy and concussed athletes. There are several findings of interest. First, the PSD features collected from frequency sub-bands played an important role in distinguishing concussed individuals. Discriminative features were observed in delta, alpha, beta and gamma frequency bands. A difference was also noted in theta frequency band. It should be pointed out that similar frequency bands were targeted in some previous EEG studies of concussion^47,72,73^. An increase in delta and theta frequency and a decrease in beta frequency were also reported by McCrea *et al*.^40^ and Slobounov *et al*.^72^. The discrimination at reported by different frequency bands can indicate significant neuronal dysfunction. According to Demos *et al*.^73^, an increase in delta frequency may indicate brain injuries, learning problems, or difficulties with cognition. The decrease in alpha band power exhibited through the analysis partially overlaps with the results reported by Thatcher *et al*. in a previously conducted mTBI based study where coherence, phase, and power analysis was performed on EEG data collected from 130 participants^43^. The decrease in alpha power exhibited by concussed athletes compared to control peers may be interpreted as a reflection of reduced cortical excitability^74^. A substantial decrease in beta and gamma power was also revealed by the analysis. Certain levels of beta waves allow easy focus and involvement in conscious thought and logical thinking, whereas a decrease in beta waves may point to poor cognition, difficulty in concentration^73^. Moreover, a movement plan based study in terms of reaction time and endpoint error reported that a decrease in beta power is correlated with higher end point error^75^. A study conducted by Kwon *et al*. demonstrated a reduced gamma power by schizophrenia patients and concluded that the deficit might reveal a less effective local neuronal synchronization to external stimuli in the thalamic sensory oscillations or in the sensory cortex^76^. A decrease in gamma power was also reported to be correlated with lower consciousness in the anesthesia study conducted by Pritchett *et al*.^77^. Several studies also reported that a decrease in gamma power is frequently related to an increase in the low-frequency range (delta frequency band) power^78,79^ and interpreted to be related to lower neuronal activity of the brain region that operates to generate behavior^80^. All these specific power increases in the slower frequency band (delta), combined with the decrease of power in faster frequency bands (alpha, beta, gamma) exhibited by concussed athletes may imply that their neurological status is not as sound as their healthy matched peers in the control group.

Though a lot of studies revealed significant differences in EEG sub-bands, there is no signature profile to indicate increase or decrease of band powers associated with concussion. That’s why the pathophysiology of concussion is considered heterogeneous and not yet completely understood. To reinforce our EEG-based functional deficits hypothesis, in an innovative approach, the PSD based analysis for each of the EEG individual frequencies was conducted. After analyzing 189 cases, *i.e*., three different trials in three different conditions (EO, EC, VT) for 21 subjects as shown in Fig. 2, it was concluded that four ranges of frequencies are more efficient in highlighting deficits following a concussion. These ranges are slow delta (1–2 Hz), slow alpha (9–10 Hz), fast beta (20–30 Hz) and fast gamma (34–39 Hz). A similar individual frequency-based analysis conducted by us on eyes closed EEG collected from a different dataset of 20 healthy and 20 immediate concussed athletes also resulted in a nearly similar range of frequencies (1–2 Hz of delta band, 8–10 Hz of the alpha band, 24–29 Hz of the beta band and 34–36 Hz range within the gamma band)^41^. To date, no individual frequency based study was conducted for concussion assessment, and more collaborative research is needed to establish a direct relationship of these frequency bins with a concussion. The decrease in alpha band frequency bins exhibited through individual frequency analysis partially overlaps with the results reported by Thatcher *et al*. in a previously conducted mTBI based study^43^. An increase in theta band frequency bins during VT task may be associated with ADHD, depression, hyperactivity, impulsivity, and inattentiveness^51^. The individual frequency-based analysis also revealed significant differences in the upper level of beta bands compared to the lower level frequency bins. Oscillatory activity in the beta band was previously reported to reflect the presence of inhibition of the process of ongoing motor task^81^.

Elgendi *et al*. demonstrated an Alzheimer disease (AD) study and reported that new optimized frequency ranges (4–7 Hz, 8–15 Hz, 19–24 Hz) resulted in better classification accuracy than the traditional frequency bands for the diagnosis of AD^82^. Similarly, if we consider the neurological deficits observed in individual frequency bins, as well as in the conventional frequency bands as a whole, the most reliable interpretation is that these deficits may be a consequence of their injury and can possibly be used as a concussion assessment index to identify the concussed athletes at the time of injury or during the post-concussion recovery period.

In the second phase of this study, a set of time-domain and nonlinear features were extracted. These features have been proven to be suitable to characterize neurological disorders like epilepsy, attention-deficit/hyperactivity disorder (ADHD) and Alzheimer disease in the literature^83^. It was hypothesized that the time domain and nonlinear feature based study could reveal new aspects and provide more information regarding the complex and chaotic nature of the EEG data. As reported by Mohammadi *et al*.^84^, quantitative measures of chaos and non-linear features are convenient descriptive tools to characterize electrophysiological abnormalities in neuropsychiatric disorders that are not evident in linear analysis. To show the effectiveness of these features for a concussion, in a similar approach to power analysis, the features were calculated for both frequency bands and individual EEG frequencies. Though the concussed athletes exhibit different values for Hjorth time domain parameters and nonlinear parameters like approximate entropy and Hurst exponent, none of the parameters showed a significant difference compared to their healthy peers for traditional EEG frequency bands. But, when the analysis was done for each frequency, it was noted that significant differences were observed for certain frequencies as shown in Fig. 3(a–c).

The observation of significantly different nonlinear features also revealed important notions about concussed athletes. The concussed athletes exhibited a decrease in Hjorth complexity and mobility. It has been reported by Pezard *et al*.^85^ that depressive subjects tend to display lower complexity than controls. Moreover, Hamida *et al*.^86,86^ reported the decreased complexity and mobility are associated with insomniac subjects. Approximate entropy quantifies the amount of regularity in data by calculating the upcoming amplitude values of the signal based on the knowledge of the preceding amplitude values^87^. Sohn *et al*.^88^ reported a significantly lower approximate entropy for a group of ADHD subjects compared to matched controls and hypothesized that the patients might not have sufficient levels of cortical activation to reach the requirements of attention-demanding tasks. Following their hypothesis, a significant decrease in approximate entropy exhibited by concussed athletes may point out that their cortical information processing is altered compared to healthy athletes. Moreover, many pathological disorder studies like schizophrenia, posttraumatic stress disorder, panic disorder, and epilepsy reported lower complexity in pathological states compared to healthy subjects^89^. The notion claimed by the authors is that the lower EEG complexity is attributed to the abnormal neural integration in the above-mentioned mental disorders^58^ and thus a lower value of ApEn demonstrated by concussed athletes in our study implies that they may still have some irregularity in their neural integration.

Another nonlinear feature with a significant difference was the Hurst exponent. Higher values of Hurst exponent indicate a stronger long-range temporal correlation of amplitude fluctuations of EEG^55^. In accordance with the result reported by Geng *et al*.^90^ in their epileptic study, a decreased Hurst exponent exhibited by concussed athletes in our study implies that the degree of anti-correlation of concussed athletes is larger than that of healthy athletes.

The most efficient frequencies indicating the deficits were found to be 1–3 Hz, 21–24 Hz, 28–30 Hz and 35–38 Hz. Among the EEG task condition, EO and VT conditions were found to be more efficient in identifying hidden deficits due to a concussion. Though conventional band base analysis revealed no significant difference between healthy and concussed athletes regarding time domain and nonlinear features, individual frequency analysis was efficacious to exhibit these hidden discrepancies. These differences at specific frequencies would remain unnoticed if only conventional frequency bands were considered. Ultimately, this study exposed the fact that EEG analysis for each frequency is equally as important as conventional bands to evaluate the neurological dysfunction following a concussion.

## Conclusions

This study suggests that EEG analysis is more sensitive compared to cognitive testing to decipher persistent sequelae of sport-related concussion. For the first time, a set of time domain and nonlinear EEG features was utilized in addition to the standard frequency band features to highlight neuronal deficits following a concussion. Also, the approach of analysis using individual frequencies of EEG was conducted for the first time to study concussion. This innovative approach combined with novel features opens a new door to interpret subtle post-concussion deficits. While no previous work was done to find the post-concussive deficits in individual frequency level, the result demonstrated a new range of frequency which is more successful to reveal the discrepancies. In sum, accumulated evidence from this study suggests that the proposed approach of EEG analysis was successful to identify that the athletes with a history of concussive injury still exhibited neurological alterations, despite reporting to be symptom-free by standard postural, visual or neurophysiological tests.

### Limitations and Future Work

Although the current manuscript has several strengths, some limitations should be considered. First, the analysis was cross-sectional, and it is always possible that some unmeasured variable may add to the current group alterations. This probability is minimalized, however, as the study groups were co-players cautiously matched for weight, height, age, years of education, and sport. Although we repeated the experiment in three separate sessions and using three different conditions, the data set is small and was limited to male athletes only. As such, the conclusions drawn from the current dataset should be used to guide similar studies on larger datasets and other age groups. However, this is an ongoing project, and we are collecting data from more participants so that more rigorous quantitative and qualitative analysis can be performed with a larger data set consisting of recordings from a large number of subjects in the future. Future work would also include applying the proposed methodology for the classification of two classes, namely healthy and concussed, to detect and predict the concussion from EEG signals for the normal and abnormal condition. Therefore, our findings will engender more comprehensive evaluations towards clinical applicability of concussion assessment for proper diagnosis and prevention through accurate RTP decision, as well as managing the treatment and rehabilitation efficacy post-concussion.

## Electronic supplementary material

Supplementary Table. EEG power deficits in terms of mean and standard deviation between healthy and concussed group for significant individual frequency bins (p-value = 0.05) for all three conditions.
