# Supplementary material for: A Novel EEG Based Spectral Analysis of Persistent Brain Function Alteration in Athletes with Concussion History
Source: Sci Rep. 2017 Dec 8;7:17221. doi: 10.1038/s41598-017-17414-x (PMC5722818; doi:10.1038/s41598-017-17414-x)
Supplement: Supplementary file 1 — Supplementary Table. EEG power deficits in terms of mean and standard deviation between healthy and concussed group for significant individual frequency bins (p-value = 0.05) for all three conditions. [file 41598_2017_17414_MOESM1_ESM.doc]

**A Novel EEG Based Spectral Analysis of Persistent Brain Function Alteration in Athletes with Concussion History**

**Tamanna T. K. Munia^1^, Ali Haider^1^, Charles Schneider^1^, Mark Romanick^2^, & Reza Fazel-Rezai^1^**

| **EC Condition** | | | **EO Condition** | | | **VT Condition** | | |
| --- | --- | --- | --- | --- | --- | --- | --- | --- |
| Significant Frequency Bins (Hz) | Healthy (μV^2^)  Mean ± SD | Concussed  (μV^2^)  Mean ± SD | Significant Frequency Bins (Hz)) | Healthy  (μV^2^)  Mean ± SD | Concussed  (μV^2^)  Mean ± SD | Significant Frequency Bins (Hz) | Healthy  (μV^2^)  Mean ± SD | Concussed  (μV^2^)  Mean ± SD |
| 1 | 4.39 ± 0.22 | 4.73 ± 0.33 | 1 | 4.49 ± 0.23 | 4.80 ± 0.35 | 1 | 4.58 ± 0.27 | 4.89 ± 0.23 |
| 2 | 3.98 ± 0.28 | 4.26 ± 0.26 | 2 | 4.03 ± 0.18 | 4.38 ± 0.24 | 2 | 4.05 ± 0.23 | 4.37 ± 0.18 |
| 3 | 3.67 ± 0.35 | 3.93 ± 0.23 | 3 | 3.78 ± 0.17 | 4.10 ± 0.23 | 3 | 3.78 ± 0.24 | 4.01 ± 0.27 |
| 9 | 3.33 ± 0.31 | 3.02 ± 0.24 | 9 | 3.24 ± 0.18 | 2.93 ± 0.24 | 6 | 3.23 ± 0.22 | 3.45 ± 0.23 |
| 10 | 3.38 ± 0.31 | 3.01 ± 0.33 | 10 | 3.23 ± 0.20 | 2.91 ± 0.27 | 7 | 3.12 ± 0.12 | 3.36 ± 0.22 |
| 15 | 2.66 ± 0.29 | 2.32 ± 0.20 | 20 | 2.46 ± 0.23 | 2.22 ± 0.19 | 9 | 3.22 ± 0.19 | 2.94 ± 0.16 |
| 16 | 2.68 ± 0.31 | 2.41 ± 0.28 | 21 | 2.44 ± 0.13 | 2.21 ± 0.15 | 10 | 3.22 ± 0.17 | 2.93 ± 0.22 |
| 17 | 2.55 ± 0.28 | 2.31 ± 0.18 | 22 | 2.39 ± 0.20 | 2.18 ± 0.22 | 19 | 2.54 ± 0.24 | 2.32 ± 0.18 |
| 18 | 2.51 ± 0.27 | 2.30 ± 0.20 | 23 | 2.32 ± 0.21 | 2.13 ± 0.14 | 20 | 2.46 ± 0.13 | 2.26 ± 0.17 |
| 20 | 2.44 ± 0.26 | 2.27 ± 0.21 | 24 | 2.25 ± 0.11 | 2.09 ± 0.18 | 21 | 2.45 ± 0.21 | 2.24 ± 0.16 |
| 21 | 2.43 ± 0.28 | 2.25 ± 0.19 | 27 | 2.11 ± 0.23 | 1.92 ± 0.12 | 22 | 2.41 ± 0.31 | 2.18 ± 0.15 |
| 22 | 2.36 ± 0.22 | 2.15 ± 0.14 | 28 | 2.07 ± 0.23 | 1.88 ± 0.16 | 23 | 2.33 ± 0.13 | 2.13 ± 0.16 |
| 23 | 2.27 ± 0.19 | 2.10 ± 0.15 | 29 | 2.05 ± 0.31 | 1.86 ± 0.11 | 24 | 2.26 ± 0.23 | 2.09 ± 0.15 |
| 24 | 2.20 ± 0.29 | 2.04 ± 0.12 | 30 | 2.01 ± 0.23 | 1.83 ± 0.20 | 25 | 2.22 ± 0.15 | 2.04 ± 0.14 |
| 28 | 2.02 ± 0.22 | 1.81 ± 0.23 | 34 | 1.90 ± 0.13 | 1.69 ± 0.19 | 26 | 2.17 ± 0.14 | 2.00 ± 0.14 |
| 29 | 2.01 ± 0.21 | 1.82 ± 0.13 | 35 | 1.88 ± 0.23 | 1.68 ± 0.19 | 27 | 2.13 ± 0.21 | 1.95 ± 0.14 |
| 30 | 1.97 ± 0.19 | 1.79 ± 0.16 | 36 | 1.84 ± 0.14 | 1.64 ± 0.12 | 28 | 2.10 ± 0.15 | 1.93 ± 0.12 |
| 35 | 1.82 ± 0.21 | 1.60±0.13 | 37 | 1.81 ± 0.23 | 1.63 ± 0.13 | 29 | 2.07 ± 0.14 | 1.89 ± 0.11 |
| 36 | 1.78 ± 0.23 | 1.56 ± 0.20 | 38 | 1.79 ± 0.14 | 1.59 ± 0.17 | 30 | 2.04 ± 0.13 | 1.86 ± 0.12 |
| 37 | 1.76 ± 0.12 | 1.55 ± 0.14 |  |  |  | 34 | 1.93 ± 0.14 | 1.75 ± 0.15 |
| 38 | 1.73 ± 0.18 | 1.53 ± 0.14 |  |  |  | 35 | 1.91 ± 0.24 | 1.72 ± 0.13 |
|  |  |  |  |  |  | 36 | 1.87 ± 0.14 | 1.68 ± 0.13 |
|  |  |  |  |  |  | 37 | 1.85 ± 0.15 | 1.67 ± 0.14 |
|  |  |  |  |  |  | 38 | 1.82 ± 0.15 | 1.65 ± 0.12 |

**Supplementary Table.** EEG power deficits in terms of mean and standard deviation between healthy and concussed group for significant individual frequency bins (*p*-value = 0.05) for all three conditions.
